# *Helicobacter pylori* eradication increases the serum high density lipoprotein cholesterol level in the infected patients with chronic gastritis: A single-center observational study

**DOI:** 10.1371/journal.pone.0221349

**Published:** 2019-08-16

**Authors:** Naoto Iwai, Takashi Okuda, Kohei Oka, Tasuku Hara, Yutaka Inada, Toshifumi Tsuji, Toshiyuki Komaki, Ken Inoue, Osamu Dohi, Hideyuki Konishi, Yuji Naito, Yoshito Itoh, Keizo Kagawa

**Affiliations:** 1 Department of Gastroenterology and Hepatology, Fukuchiyama City Hospital, Fukuchiyama-city, Kyoto, Japan; 2 Department of Molecular Gastroenterology and Hepatology, Graduate School of Medical Science, Kyoto Prefectural University of Medicine, Kyoto, Japan; National Cancer Center, JAPAN

## Abstract

**Background:**

Extra-gastric manifestation *of Helicobacter pylori* infection involves systemic inflammation, which results in the production of several cytokines. Only a few clinical trials have investigated the effect of *H*. *pylori* eradication therapy on lipid metabolism in the infected patients with chronic gastritis. We aimed to evaluate the effect of *H*. *pylori* eradication therapy on lipid metabolism in a Japanese population with chronic gastritis.

**Methods:**

One hundred and sixty-three patients with *H*. *pylori*-associated chronic gastritis were enrolled in this study between June 2015 and March 2017. They underwent *H*. *pylori* eradication therapy; the effects of the therapy were assessed by the urea breath test performed at least 4 weeks after the therapy. After confirming *H*. *pylori* eradication, the health screening examination was repeated between May 2016 and August 2018. The clinical parameters were compared before and after the administration of the eradication therapy.

**Results:**

The mean age of the enrolled patients was 56.7 years, and the mean follow-up duration was 514.7 days. Weight, body mass index, and obesity index were significantly increased post-eradication therapy compared to those pre-eradication therapy. White blood cell and platelet counts were significantly decreased, and high density lipoprotein cholesterol (HDL) level was significantly increased (*P* = 0.001*)*, while low density lipoprotein cholesterol (LDL), total cholesterol, and triglycerides levels were not altered significantly. Hence, the LDL/HDL ratio was significantly decreased.

**Conclusions:**

This study reported that *H*. *pylori* eradication therapy increase the HDL levels in the infected patients with chronic gastritis. Hence, the LDL/HDL ratio, which is used to evaluate the risk of atherosclerosis, was significantly decreased post-eradication therapy compared to that pre-eradication therapy.

## Introduction

*Helicobacter pylori* (*H*. *pylori*) is a gram-negative bacteria that causes chronic gastritis, peptic ulcer, and gastric cancer.[1, 2] *H*. *pylori* eradication therapy can prevent peptic ulcer recurrence and may possibly result in decreasing the incidence of gastric cancer.[3, 4] In contrast, previous studies reported that *H*. *pylori* infection can cause extra-gastrointestinal (GI) disease, including nonalcoholic fatty liver disease, dyslipidemia and coronary heart disease.[5–8] *H*. *pylori* infection causes chronic and persistent inflammation, which results in the production of cytokines, including tumor necrosis factor-α, interleukin (IL)-6 and IL-8.[9–11] The previous reports revealed that *H*. *pylori* infection may worsen serum lipid levels through long-term chronic inflammation caused by *H*. *pylori*.[6–8, 12–18] In addition, *H*. *pylori* eradication therapy may potentially improve the lipid profiles by inhibiting the release of inflammatory cytokines.[19–24] However, in some reports, the study subjects were confined to patients with peptic ulcers or functional dyspepsia.[19, 24, 25] A few clinical trials have investigated whether *H*. *pylori* eradication therapy improves lipid metabolism in patients with *H*. *pylori*-associated chronic gastritis.[21, 23]

In Japan, eradication therapy for *H*. *pylori*-associated chronic gastritis has been included in the national health insurance program since February 2013.[26] Subsequently, *H*. *pylori*-infected patients without peptic ulcer or early gastric cancer have increasingly undergone eradication therapy. The established insurance system may have a potential role in decreasing the incidence of gastric cancer.[26] However, the mechanism of *H*. *pylori* eradication and its effect on extra-gastric manifestations in patients with chronic gastritis remains controversial.

Therefore, it is important to investigate whether *H*. *pylori* eradication therapy for chronic gastritis may significantly alter lipid metabolism in the infected patients. This study aimed to evaluate the effect of *H*. *pylori* eradication therapy on lipid metabolism in the infected patients with chronic gastritis.

## Materials and methods

### Patients

The patients who underwent the health screening examination between June 2015 and March 2017 were analyzed. When the upper GI endoscopic examination indicated the presence of an *H*. *pylori* infection, the serum IgG anti-*H*. *pylori* test was performed based on patient preferences. Based on the manufacturer’s instructions, *H*. *pylori* infection was defined as the presence of a serum IgG antibody level of more than 10 IU/mL. When both endoscopic findings and the serum IgG test indicated *H*. *pylori* infection, the patients underwent eradication therapy. The patients with active gastroduodenal ulcers and gastric cancer and those undergoing treatment for hyperlipidemia or failure of prior eradication were excluded. Finally, a total of 163 patients with successful eradication of *H*. *pylori* were enrolled in this study and underwent a health screening examination again between May 2016 and August 2018.

The first-line eradication therapy comprised administration of a proton pump inhibitor or vonoprazan, amoxicillin, and clarithromycin twice daily for 1 week. The effects of the eradication therapy were assessed by performing the urea breath test at least 4 weeks after the therapy. A Δ13C level of less than 2.5‰ indicated successful eradication of *H*. *pylori*, while that of more than 2.5‰ was determined as a failure of *H*. *pylori* eradication.[27] When the urea breath test results indicated failure of *H*. *pylori* eradication, the second-line eradication therapy comprising administration of a proton pump inhibitor or vonoprazan, amoxicillin, and metronidazole twice daily for 1 week was initiated. The clinical parameters recorded before and after the administration of the eradication therapy were compared.

This study was conducted in accordance with the ethical guidelines of the Declaration of Helsinki, and the study protocol was approved by the ethics committee of the Fukuchiyama City Hospital. All data were fully anonymized before we accessed them, and the ethics committee of the Fukuchiyama City Hospital approved a waiver of informed consent because anonymized clinical data were used in this study.

### Data collection

The serum IgG anti-*H*. *pylori* test was performed using an enzyme-linked immunosorbent assay (Eiken Chemical Co. Ltd, Tokyo, Japan). A serum IgG antibody level of more than 10 IU/mL was defined as the presence of *H*. *pylori* infection. The body mass index (BMI), obesity index, and body fat percentage were automatically calculated using the Tanita DC-270A analyzer (Tanita Corporation, Tokyo, Japan). All patients underwent blood and biochemical tests twice, before and after *H*. *pylori* eradication. The biochemical tests were performed in the morning after overnight fasting.

The following factors were evaluated for all the patients; white blood cell (WBC) count, red blood cell (RBC) count, hematocrit (Hct) volume, mean corpuscular volume, mean corpuscular hemoglobin, mean corpuscular hemoglobin concentration, platelets (Plt) count, and levels of hemoglobin (Hb), total cholesterol (T-cho), high density lipoprotein cholesterol (HDL), low density lipoprotein cholesterol (LDL), triglycerides, uric acid, aspartate aminotransferase, alanine aminotransferase, blood urea nitrogen, creatinine, fasting plasma glucose, and hemoglobin A1c.

### Statistical analysis

The continuous variables were expressed as means and standard deviations (SD). The categorical variables were expressed as numbers and percentages. The Wilcoxon signed-rank test was used to compare the values of parameters before and after the administration of the eradication therapy. *P* values <0.05 were considered statistically significant. All statistical analyses were performed using IBM SPSS version 25.0 for Windows (IBM SPSS, Chicago, IL, USA).

## Results

The characteristics of the *H*. *pylori*-eradicated patients are shown in Table 1. The mean age of the enrolled patients was 56.7 years. The mean anti-*H*. *pylori* IgG antibody titer in the serum was 47.5 U/ml. The mean follow-up duration was 514.7 days.

**Table 1:** Characteristics of the *H*. *pylori*-eradicated subjects.

No. patients	163	
Age	56.7	±11.6
Sex, n(%)		
Male	86	(52.8)
Female	77	(47.2)
Alcohol consumption, n(%)		
<20g/day	110	(67.5)
>20g/day and <40g/day	32	(19.6)
>40g/day and <60g/day	10	(6.1)
>60g/day	3	(1.8)
Unknown	8	(4.9)
Smoking, n(%)	22	(13.5)
Medical history, n(%)		
Hypertension	30	(18.4)
Diabetes mellitus	5	(3.1)
Hyperuricemia	5	(3.1)
Liver disease	2	(1.2)
Benign prostatic hyperplasia	6	(3.7)
Gynecologic disease	13	(8.0)
Serum anti-*H*. *pylori* IgG antibody (U/ml)	47.5	±29.7
Prevalence of gastroduodenal ulcer scar, n(%)	26	(16.0)
*H*. *pylori* eradication therapy		
First-line therapy, n(%)	137	(84.0)
Second-line therapy, n(%)	26	(16.0)
Follow-up duration	514.7	±199.8

The effect of *H*. *pylori* eradication is shown in Table 2. Weight, obesity index and BMI significantly increased post-eradication therapy compared to the pre-eradication therapy values. The blood test results showed a significant decrease in the WBC and Plt counts, with no remarkable change in the RBC counts. No significant changes were observed in the liver function, renal function or glucose metabolism.

**Table 2:** Effect of *H*. *pylori* eradication.

	Baseline		After		*p* value
Body constitution					
Height (cm)	162.9	±8.1	162.9	±8.2	0.914
Weight (kg)	59.6	±13.8	60.1	±13.9	0.001
BMI (kg/m^2^)	22.4	±4.5	22.6	±4.5	0.003
Obesity index	1.8	±20.5	2.7	±20.5	0.005
Body fat percentage (%)	25.9	±7.2	26.2	±7.1	0.167
Waist (cm)	81.5	±9.9	82.1	±10.3	0.088
Blood test					
WBC count (×10^3^/μL)	5372.1	±1359.4	4989.2	±1371.1	<0.001
RBC count (×10^6^/μL)	461.9	±37.5	461.4	±38.2	0.607
Hb (g/dL)	13.9	±1.2	13.9	±1.2	0.536
Hct (mg/dL)	42.1	±3.2	42.1	±3.1	0.745
MCV (fl)	91.3	±5.2	91.5	±4.6	0.638
MCH (pg)	30.1	±2.1	30.2	±1.8	0.368
MCHC (%)	33	±0.9	32.9	±0.8	0.289
Plt count (×10^3^/μL)	24.3	±5.9	23.5	±5.5	0.001
Biochemical test					
AST (mg/dL)	22.5	±6.3	23.3	±7.0	0.205
ALT (mg/dL)	20	±9.1	21.1	±10.7	0.393
BUN (mg/dL)	14.4	±3.6	14.3	±3.5	0.516
Cre (mg/dL)	0.72	±0.17	0.73	±0.18	0.949
UA (mg/dL)	5.21	±1.38	5.25	±1.38	0.505
FPG (mg/dL)	99.8	±19.9	99.9	±23.9	0.492
HbA1c (%)	5.77	±0.86	5.79	±0.88	0.160

BMI, body mass index; WBC, white blood cell; RBC, red blood cell; Hb, hemoglobin; Hct, hematocrit; MCV, mean corpuscular volume; MCH, mean corpuscular hemoglobin; MCHC, mean corpuscular hemoglobin concentration; Plt, platelets; AST, aspartate aminotransferase; ALT, alanine aminotransferase; BUN, blood urea nitrogen; Cre, creatinine; UA, uric acid; FPG, fasting plasma glucose; HbA1c, hemoglobin A1c.

**Fig 1:**
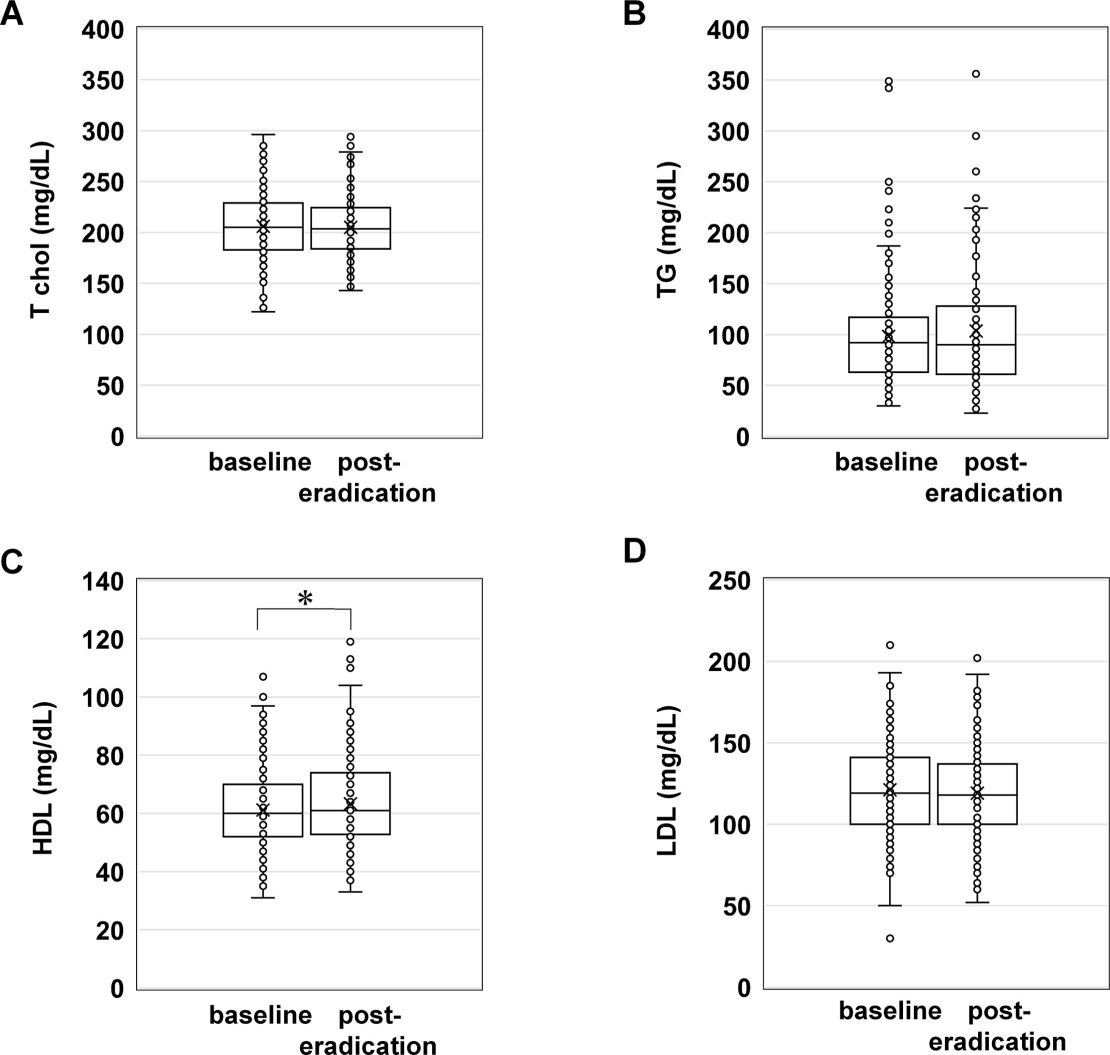
Lipid profiles at baseline and post-eradication therapy. (A) Box plot showing serum the T-cho levels at baseline and post-eradication therapy. (B) Box plot showing the serum TG levels at baseline and post-eradication therapy. (C) Box plot showing the serum HDL levels at baseline and post-eradication therapy. *P < 0.01. (D) Box plot showing the serum LDL levels at baseline and post-eradication therapy. Scatter dot plots show the measured serum levels. The middle line represents the median. The symbol “x” in the box plot represents the mean. T-cho, total cholesterol; TG, triglycerides; HDL, high density lipoprotein cholesterol; LDL, low density lipoprotein cholesterol.

**Fig 2:**
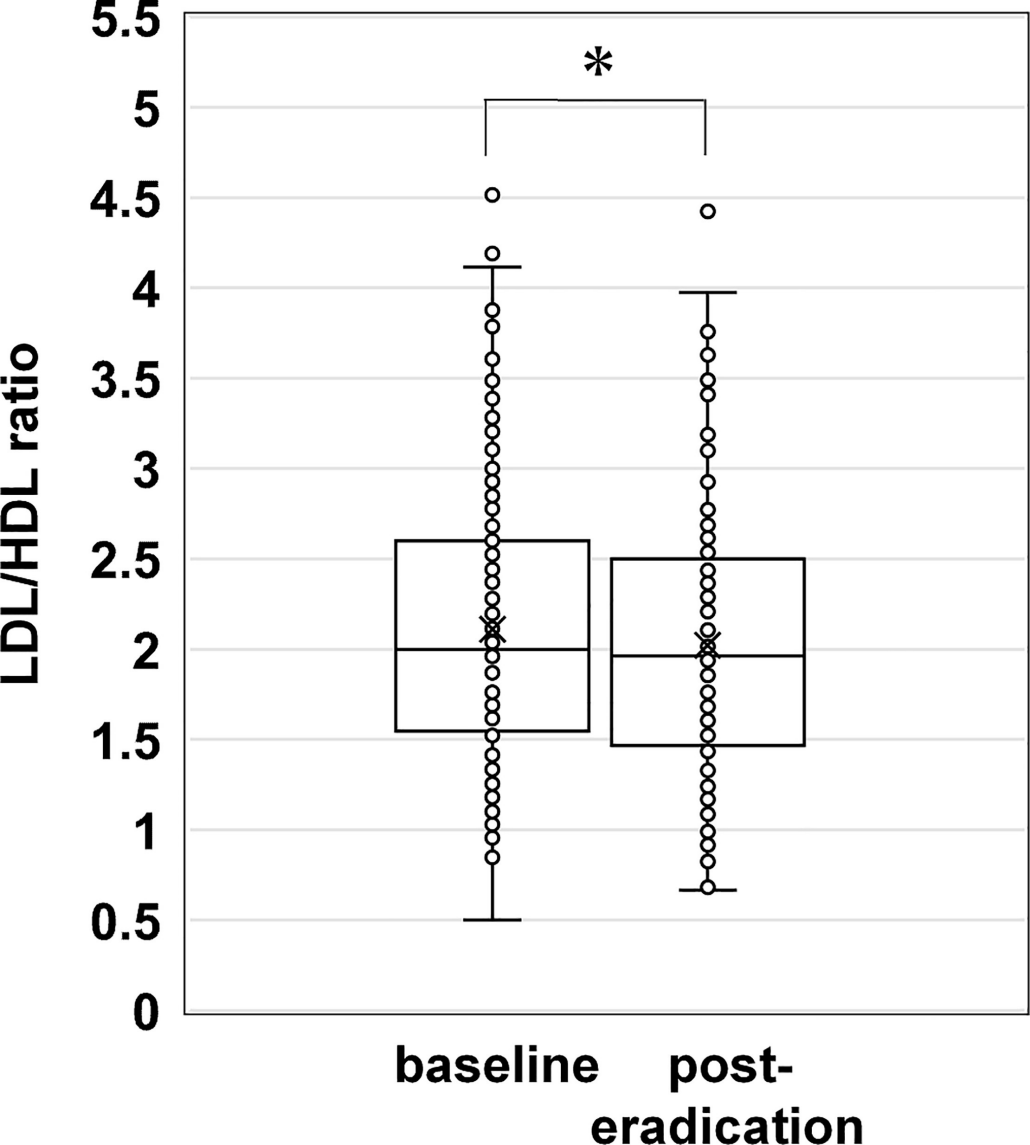
Low density lipoprotein / high density lipoprotein cholesterol ratios at baseline and post-eradication therapy. Box plot showing the serum LDL/HDL ratios at baseline and post-eradication therapy. *P < 0.01. Scatter dot plots show the measured serum levels. The middle line represents the median. The symbol “x” in the box plot represents the mean. HDL, high density lipoprotein cholesterol; LDL, low density lipoprotein cholesterol.

In the lipid profile, the HDL level (61.2 ± 14.7 mg/dL at baseline versus 63.3 ± 15.8 mg/dL at post-eradication therapy, *P* < 0.01) was significantly increased, while the T-cho (206.0 ± 32.5 mg/dL versus 205.1 ± 30.8 mg/dL), LDL (121.2 ± 28.7 mg/dL versus 119.0 ± 27.6 mg/dL), and TG (98.1 ± 50.9 mg/dL versus 103.5 ± 58.0 mg/dL) levels did not change significantly (Fig 1). Hence, the LDL/HDL (2.11 ± 0.75 mg/dL at baseline versus 2.02 ± 0.76 mg/dL at post-eradication therapy, *P* < 0.01) ratio was significantly decreased post-eradication therapy compared to that pre-eradication therapy (Fig 2).

## Discussion

In the present study, we noted a significant increase in the weight, BMI, and obesity index of patients with *H*. *pylori*-associated chronic gastritis, approximately 1.5 years after the administration of the *H*. *pylori* eradication therapy. In addition, *H*. *pylori* eradication induced a decrease in the WBC and Plt counts. With respect to the lipid profiles, the HDL level was significantly increased, while the LDL/HDL ratio was significantly decreased. The results show that *H*. *pylori* eradication therapy may prevent the development of arteriosclerosis by the regulation of serum lipid concentrations, especially the HDL levels.

Inflammatory cytokines may have an essential role in dyslipidemia and arteriosclerosis.[8, 28, 29] In Japan, the national health insurance system was originally established for *H*. *pylori* eradication therapy to prevent chronic gastritis. Since an increasing number of patients with chronic gastritis undergo *H*. *pylori* eradication, it is important to assess the systemic effects caused by the eradication. However, little is known, especially, regarding the alterations in the lipid profiles after administration of the *H*. *pylori* eradication therapy in the infected patients with chronic gastritis.[21, 23]

In accordance with the previous reports, weight and BMI significantly increased after administration of *H*. *pylori* eradication therapy.[30–33] Lane et al. confirmed that BMI and weight significantly increased on administration of *H*. *pylori* eradication therapy not only in the Japanese patients, but also in the European population because of improvement in dyspepsia.[33] In addition, the WBC and Plt counts significantly decreased after administration of the *H*. *pylori* eradication therapy. The WBC count was reported to increase in proportion to the ratio of the *H*. *pylori* infection.[34] These findings suggest that chronic inflammation caused due to *H*. *pylori* infection increased both the WBC and Plt counts. The eradication therapy could eliminate chronic inflammation, which results in decreased WBC and Plt counts. Kanbay et al. reported that CRP levels were also decreased on administration of the *H*. *pylori* eradication therapy [20]. This report provided additional evidence that the eradication therapy could reduce systemic inflammation.

This study reported that the HDL level was significantly increased after the administration of the eradication therapy, as reported in previous studies.[19–21, 23, 24] Systemic inflammation was previously proven to alter the composition of HDL protein and lipid, and decrease the HDL levels.[35, 36] In addition, inflammation could transform HDL into a dysfunctional condition. These observations suggested that inflammatory cascades induced by *H*. *pylori* resulted in a decrease in the HDL levels. However, the eradication therapy restored the serum HDL level by an improvement in the inflammatory status. Unlike the results of our study, Elizalde et.al [37] reported that the HDL levels significantly increased in *H*. *pylori* infected patients, irrespective of the administration of the eradication therapy, when the HDL level was evaluated at baseline and 3 months after the administration of the eradication therapy. They suggested that the increase in the HDL level may be due to improvement in dyspepsia and lifestyle and, not due to the *H*. *pylori* eradication therapy itself. The difference may be because treatment with antacids could inhibit *H*. *pylori*-induced chronic gastritis in some patients who did not undergo eradication therapy but underwent treatment with antacids, with follow-up periods shorter than that in our study. [38] Obesity was reportedly related to disturbances in the lipid profiles [39]; however, administration of the *H*. *pylori* eradication therapy caused both weight gain and improvement of lipid metabolism in this study. This discrepancy may be because the regulation of the HDL levels in the *H*. *pylori*-eradicated patients was more strongly influenced by the suppression of systemic inflammation than by weight gain. However, the mean follow-up duration in this study was approximately 1.5 years. In further studies, we should investigate the effect of *H*. *pylori* eradication on lipid profiles and weight over a longer follow-up period.

In general, HDL has an atheroprotective role, while LDL has an atherosclerotic role. Moreover, the LDL/HDL ratio has been recently considered a better predictive parameter compared with the HDL and LDL levels alone, for the assessment of the severity of coronary plaque or carotid atherosclerosis.[40–42] Our findings revealed that the LDL/HDL ratio significantly decreased following the administration of the eradication therapy. The results suggested that *H*. *pylori* eradication may possibly contribute to anti-atherogenic properties.

The present study has several limitations. First, this was a single center and retrospective study. Second, due to the small sample size, it is difficult to generalize the results of this study. Our results must be confirmed in a large-scale population validation analysis, such as a study with moer than 500 cases. If possible, meta-analyses may be recommended to confirm the effect of *H*. *pylori* eradication on lipid profiles. Third, the mean follow-up period was approximately 1.5 years. Therefore, it is unclear whether our findings can be extrapolated to a longer follow-up period. Our results must be validated in a prospective clinical trial allowing for a longer observation period. Fourth, our study did not include the *H*. *pylori*-negative patients as a control group. Thus, we could not evaluate the changes in lipid metabolism in *H*. *pylori*-negative patients; however, we believe that our results provide insights into the lipid metabolism of *H*. *pylori*-positive patients.

In conclusion, we showed that administration of the *H*. *pylori* eradication therapy increased the HDL levels in the infected patients with chronic gastritis. In addition, a decrease in the LDL/HDL ratio suggests that *H*. *pylori* eradication may possibly play an anti-atherogenic role in the infected patients with chronic gastritis.

## Supporting information

S1 Table Summary of the published data on the changes in the lipid profiles at baseline and post-eradication therapy. (DOCX)
