# Supplementary material for: Helicobacter pylori eradication increases the serum high density lipoprotein cholesterol level in the infected patients with chronic gastritis: A single-center observational study
Source: PLoS One. 2019 Aug 16;14(8):e0221349. doi: 10.1371/journal.pone.0221349 (PMC6697333; doi:10.1371/journal.pone.0221349)
Supplement: S1 Table — (DOCX) [file pone.0221349.s001.docx]

***Helicobacter pylori* eradication increases the serum high density lipoprotein cholesterol level in the infected patients with chronic gastritis: A single-center observational study**

Naoto Iwai^1, 2^, Takashi Okuda^1^, Kohei Oka^1^, Tasuku Hara^1^, Yutaka Inada^1^, Toshifumi Tsuji^1^, Toshiyuki Komaki^1^, Ken Inoue^2^, Osamu Dohi^2^, Hideyuki Konishi^2^, Yuji Naito^2^, Yoshito Itoh^2^, Keizo Kagawa^1, 2^

^1^Department of Gastroenterology and Hepatology, Fukuchiyama City Hospital, Fukuchiyama-city, Kyoto, Japan.

^2^Department of Molecular Gastroenterology and Hepatology, Graduate School of Medical Science, Kyoto Prefectural University of Medicine, Kyoto, Japan.

**Supporting information**

**S1 Table:** Summary of the published data on the changes in the lipid profiles at baseline and post-eradication therapy.

| Patients | Number of patients (n) | Follow-up period (weeks) | Change in  T chol levels | Change in HDL levels | Change in LDL levels | Change in  TG levels | References |  |
| --- | --- | --- | --- | --- | --- | --- | --- | --- |
| Duodenal ulcer | 87 | 52 | 21.0 | 6.0 | 11.0 | 20.0 | Scharnagl H et al. [19] |  |
| *H. pylori* infection | 57 | 8 | -3.0 | 2.0 | -1.1 | 1.7 | Kanbay M et al. [20] |  |
| *H. pylori* infection | 529 | 104 | -5.1 | 1.6 | -6.4 | 12.5 | Nam SY et al. [21] |  |
| *H. pylori* infection | 185 | 104 | 3.0 | 1.4 | -0.9 | 1.3 | Adachi K et al. [22] |  |
| Functional dyspepsia | 91 | 12 | -8.6 | 2.7 | -6.2 | -5.5 | Mokhtare M et al. [24] |  |
| Peptic ulcer | 50 | 52 | 18.0 | unknown | unknown | 48.8 | Kamada T et al. [25] |  |
| *H. pylori* infection | 368 | 12 | 5.0 | 2.3 | 1.5 | 3.8 | Elizalde JI et al. [36] |  |
| Changes in the lipid profiles were calculated as follows: mean serum levels post-eradication therapy - mean serum levels at baseline.  HDL, high density lipoprotein cholesterol *H. pylori, Helicobacter pylori*; LDL, low density lipoprotein cholesterol; T-chol, total cholesterol; TG, triglyceride | | | | | | | | |
